# Volume of parasagittal dura is associated with blood markers of systemic inflammation

**DOI:** 10.1007/s00701-025-06682-6

**Published:** 2025-09-25

**Authors:** Paulina Eide, Erik Melin, Geir Ringstad, Per Kristian Eide, Angelika Sorteberg

**Affiliations:** 1 Faculty of Medicine, University of Oslo, Oslo, Norway; 2 Dept. of Radiology, Østfold Hospital Trust, Grålum, Norway; 3 Institute of Clinical Medicine, Faculty of Medicine, University of Oslo, Oslo, Norway; 4 Dept. of Radiology, Oslo University Hospital-Rikshospitalet, Oslo, Norway; 5 K.G. Jebsen Centre for Brain Fluid Research, University of Oslo, Oslo, Norway; 6 Dept. of Geriatrics and Internal Medicine, Sorlandet Hospital, Arendal, Norway; 7 Deptartment of Neurosurgery, Oslo University Hospital-Rikshospitalet, Oslo, Norway

**Keywords:** Inflammation, Inflammatory markers, C-reactive protein, Erythrocyte volume fraction, Hemoglobin, Parasagittal dura, Cerebrospinal fluid

## Abstract

**Background:**

The parasagittal dura (PSD) has been hypothesized to act as a neuro-immunological hub, though evidence in favor of the hypothesis is limited. The present study explored whether there is any association between volume and function of PSD and blood markers that may be indicative of systemic inflammation.

**Methods:**

The patient material included 76 subjects examined with intrathecal contrast-enhanced magnetic resonance imaging (MRI) where the contrast served as a cerebrospinal fluid (CSF) tracer. They were dichotomized into a control- (Ctrl) and a CSF-group. We investigated the correlations between blood markers indicative of systemic inflammation and the volume of PSD, enrichment of tracer in the PSD, and blood-measures of CSF clearance. The blood markers included C-reactive protein (CRP), erythrocyte volume fraction (EVF), hemoglobin (Hb), platelet count, neutrophiles, lymphocytes, and the neutrophil-to-lymphocyte ratio as well as the platelet-to-lymphocyte ratio. Assessment of subjective sleep quality also was included in the analysis.

**Results:**

The main finding was that higher CRP indicative of systemic inflammation was correlated with lower volume of PSD. There was an association between lower EVF and Hb levels and lower volume of PSD, but this finding could be explained by confounders such as age, sex and disease. Confounders also explained a correlation between EVF and CSF clearance variables. Impaired subjective sleep quality was associated with markers of systemic inflammation, but not with PSD volume.

**Conclusion:**

Higher CRP was correlated with lower volume of PSD suggesting that the size of this anatomical structure associates with systemic inflammation. Further studies are needed to determine whether PSD volume could serve as an imaging marker of systemic inflammation.

**Supplementary Information:**

The online version contains supplementary material available at 10.1007/s00701-025-06682-6.

## Introduction

The brain is covered by three layers of meninges; the pia mater located at the glia limitans, the arachnoid membrane covering the subarachnoid space, and the dura mater. The arachnoid and dura membranes are considered continuous via the dura border cell layer consisting of dura border cells and arachnoid barrier cells [11]. In neuroscience, the role of the meninges in brain disease received limited attention until breakthrough discoveries in 2015 demonstrating functional lymphatic vessels in the dura mater capable of draining substances in the cerebrospinal fluid (CSF) to extracranial lymph nodes [1, 21]. These discoveries further created renewed interest in the function of brain meninges [4]. The dura mater is emerging as an immunological site, and meningeal immune activation may modify dura clearance function [5]. These observations may have relevance for neurodegenerative diseases. For example, in Alzheimer’s disease, failure in the meningeal clearance function of amyloid-β and tau may participate in the disease evolvement [5], and in Parkinson’s disease, failure of meningeal clearance function may be a contributor to the cerebral aggregation of α-synuclein that characterizes this disease [7].

Therefore, an intriguing question is whether, and how systemic inflammation is linked to meningeal clearance function in humans. This question has been addressed in experimental animal studies [14, 25, 30], but translation to humans is lacking. It is well established that inflammation plays an important role in several brain diseases, both acute infections and chronic inflammatory diseases [6]. Since inflammatory responses and processes occur in several brain diseases, it may be anticipated an association between systemic inflammation and morphological and functional measures of brain structures.

The parasagittal dura (PSD) that is located along the superior sagittal sinus was found to be enriched by a CSF tracer administered intrathecally [28], suggesting passage of solutes between the subarachnoid space and the interior of the dura. In clearance phase of the CSF tracer 48 h after injection, there was a positive correlation between tracer enrichment in PSD and the volume of PSD, indicating that increased PSD volume was associated with decreased clearance of tracer [23]. Others reported that in older patients with Alzheimer’s disease, increased volume of PSD was associated with increased global cerebral amyloid-β burden assessed by positron emission tomography (PET) [31]. It was proposed that the PSD is a possible neuroimmune interface [23]. If so, we might hypothesize that morphological measures of PSD associate with inflammatory markers in the blood, and perhaps meningeal clearance capacity. C-reactive protein (CRP) is an established non-specific biomarker for inflammation that shows increased levels during ongoing inflammation [18]. Different from CRP, both hemoglobin (Hb) and erythrocyte volume fraction (EVF) may decrease during inflammation. For instance, decreased levels of Hb and EVF are seen in patients with anemia caused by chronic inflammation disorders, for example inflammatory bowel disease [33].

Given this background, the primary research question of the present study was whether the volume of PSD correlates with blood markers indicative of systemic inflammation. The secondary research questions were whether blood markers suggestive of inflammation are associated with the clearance function of brain meninges and functional indices of CSF clearance function. Finally, we asked whether the PSD volume and inflammatory markers associate with subjective sleep quality, since chronic sleep disturbances are associated with systemic inflammation [16].

## Material and methods

### Study population

The present patient cohort was retrieved from a data material of patients who underwent intrathecal contrast-enhanced magnetic resonance imaging (MRI, or glymphatic MRI, gMRI), and in whom segmentation of PSD volume had been performed [23]. The MRI had been undertaken on clinical indication as part of a diagnostic work-up within the Department of Neurosurgery, Oslo University Hospital-Rikshospitalet, Oslo, Norway. Patients with contrast allergy were not eligible for inclusion, neither were patients < 18 years old.

### Measures of inflammation

The study focused on blood markers that in a non-specific way may associate with systemic inflammation: C-reactive protein (CRP), hemoglobin (Hb), erythrocyte volume fraction (EVF), platelet count (PLT), neutrophiles (Neu), lymphocytes (Lymph), and the Neutrophil-to-Lymphocyte ratio (NLR) as well as the Platelet-to-Lymphocyte ratio (PLR). Blood samples were obtained in the morning when the patient underwent gMRI.

### Volume of PSD

**Fig. 1:**
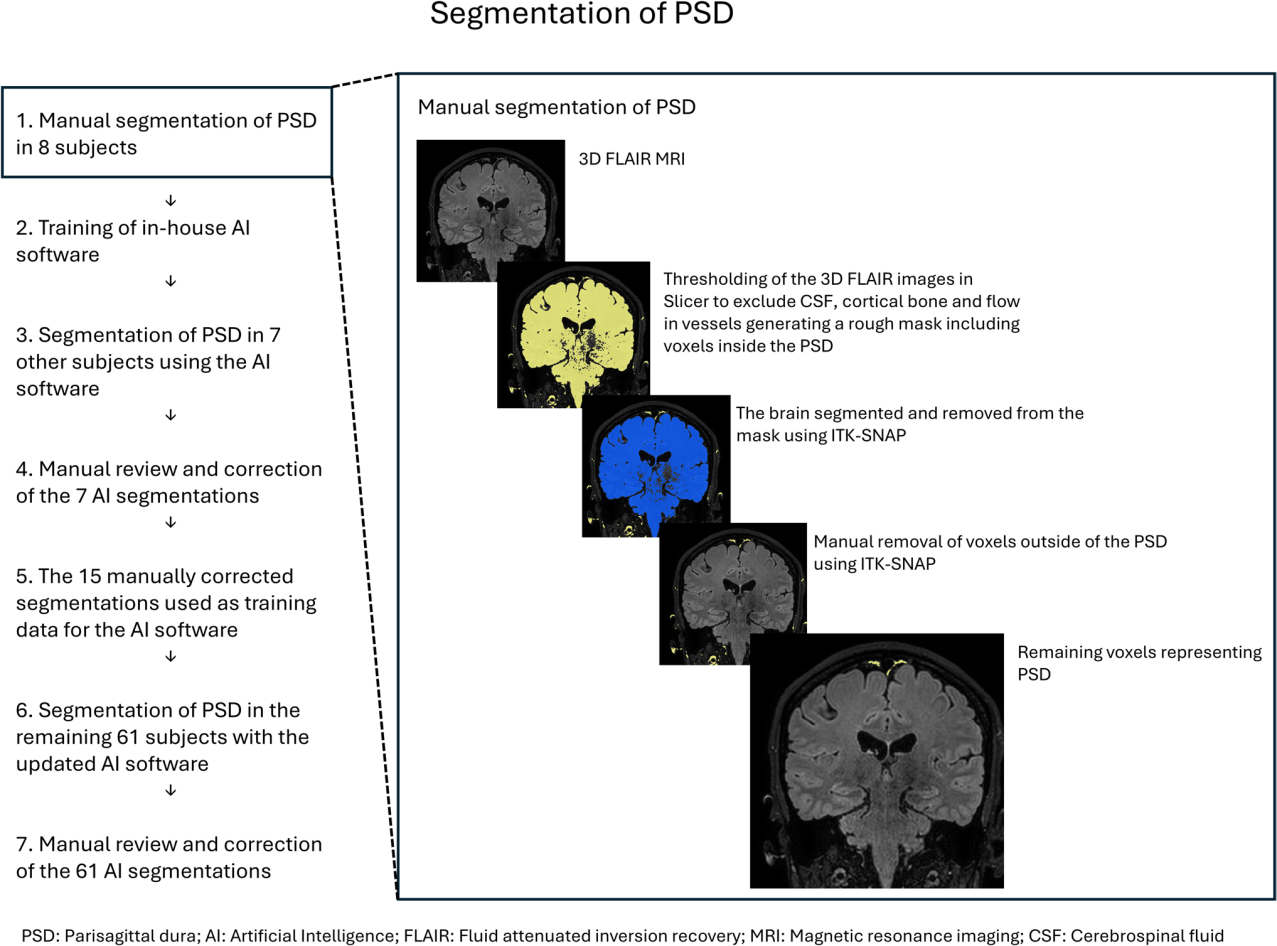
Segmentation of the parasagittal dura (PSD). We used a combination of manual segmentation with the aid of AI segmentation

The procedure of segmenting PSD for assessing PSD volume is shown in Fig. 1. In short, the FLAIR sequences were utilized for PSD segmentation. We defined PSD as the region exhibiting intermediate to high signal intensity adjacent to the superior sagittal sinus (SSS), extending from the SSS's most anterior aspect to the confluence of sinuses [28].

Manual PSD segmentation was performed on eight subjects by a neuroradiologist with nine years of clinical experience (EM). First, Slicer (version 4.11.20200930) [8] was used to threshold the images to remove fluid-attenuated CSF and signal from flowing blood and create a rough segmentation of PSD. Subsequently, adjacent structures like brain tissue, large vessels and bone marrow were Manually excluded from the rough segmentation using ITK-SNAP version 3.8.0 (http://www.itksnap.org) [34]. An artificial intelligence (AI) application utilizing a two-dimensional U-Net [29] convolutional architecture was developed using this training dataset and applied to segment PSD in an additional seven subjects. The U-Net architecture was selected due to its demonstrated performance with limited training datasets. The resulting seven AI-generated segmentations underwent manual revision in ITK-SNAP by the same neuroradiologist (EM) and served as additional training data for the AI system. The final sixty-one subjects were processed using this refined AI model, with all outputs undergoing manual review and correction when necessary. The AI model served to enhance segmentation efficiency; however, formal accuracy assessment of the AI model was not conducted.

**Fig. 2:**
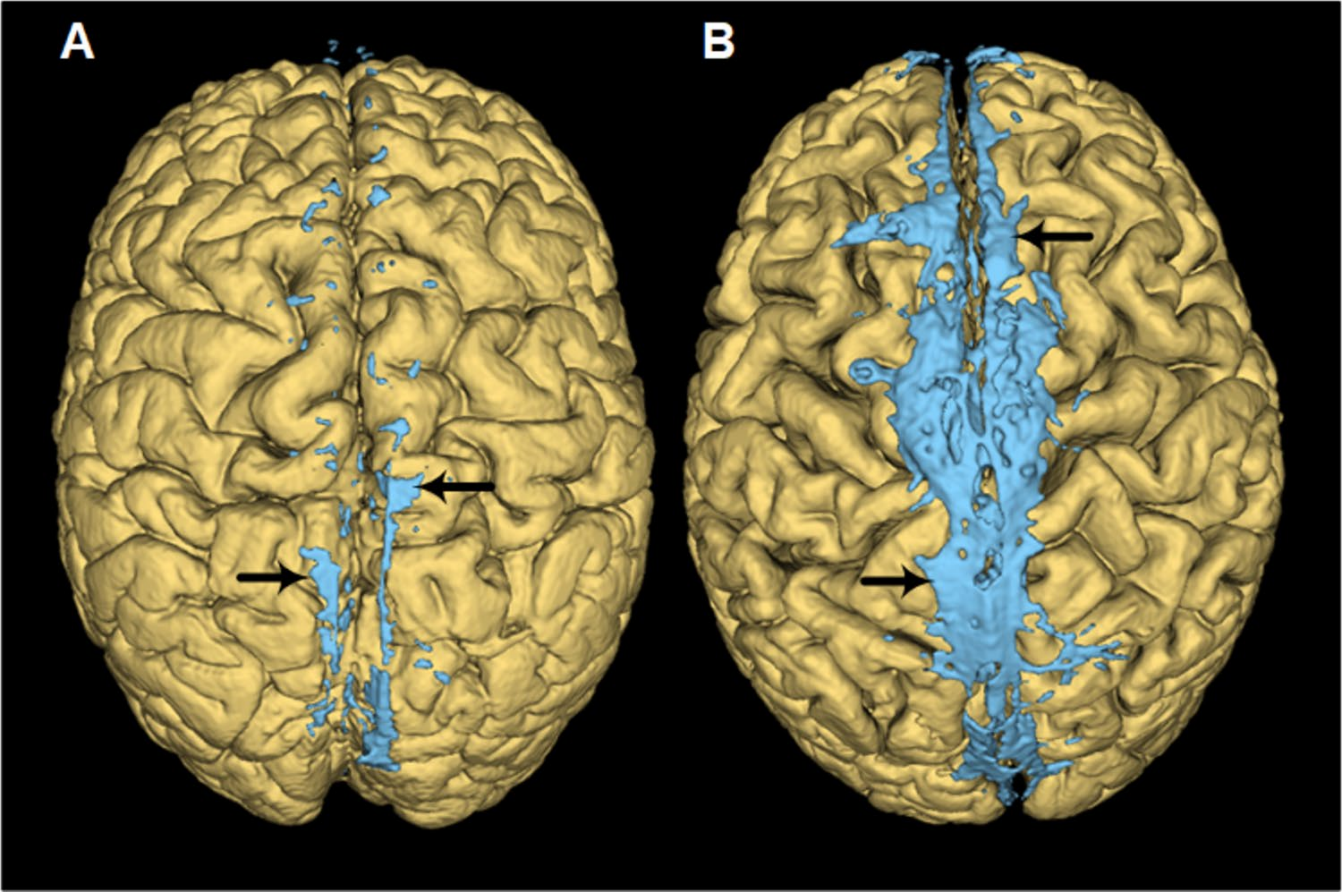
The volume of parasagittal dura (PSD) shows marked inter-individual variability. 3D images of PSD from (**A**) a patient with PSD of small volume and (**B**) another patient with larger volume PSD. PSD is depicted by blue color and indicated by the black arrows

The PSD of two patients is shown in Fig. 2.

### PSD clearance function

The enrichment of an intrathecal CSF tracer in PSD was quantified as previously described [23]. For this purpose, T1-weigthed scans were performed at different predefined time points before and (3, 6, 24 and 46 h) after intrathecal injection of an MRI contrast agent (0.5 ml gadobutrol; Gadovist®, 1 mmol/ml; Bayer AB, GE), serving as a CSF tracer. The percentage increase in tracer enrichment at PSD compared to pre-injection was used as a measure of PSD clearance function. We examined the correlation between the volume of PSD and tracer enrichment in the PSD at all time points.

### Blood-test-based measures of meningeal clearance function

As a proxy of meningeal clearance function, we retrieved information about CSF clearance to blood from a pharmacokinetic model [13]. The method has previously been described in detail [13]. In the present study, the following pharmacokinetic variables were correlated with the markers of inflammation: Half-life of absorption, i.e., the time (hours) for half the amount of tracer in the CSF is cleared to blood. Time (hours) to maximum concentration of tracer in blood. Maximum concentration of tracer in blood (µM). Area under curve (µM hour) for concentrations of tracer in blood.

### Information about symptoms

Information about subjective sleep quality was based on the Pittsburgh Sleep Quality Index (PSQI) [3], which is a commonly used tool for assessing subjective sleep quality over a time interval of one month. It was developed by Buysse et al. [3] to measure sleep quality characteristics. We applied a Norwegian translation of the questionnaire [26]. The PSQI questionnaire consists of 25 questions where 19 questions are processed into a global score and seven domains: Sleep duration, sleep disturbance, sleep onset latency, daytime dysfunction, habitual sleep efficiency, subjective sleep quality and use of sleeping medication [3]. Each domain is scored from 0 to 3; the global scores have a range from 0 to 21, higher scores indicating poor sleep quality. The patient’s response to the self-reported questionnaire was based on their sleep quality during the recent months. The PSQI scores were dichotomized into good or poor sleep quality; a global PSQI score ≤ 5 is considered as indicative of good sleep quality [24]. Impaired sleep quality was defined as a PSQI global score > 5. Accordingly, good and poor sleepers refer to general sleep quality.

### Statistical methods

For statistical analyses, SPSS (version 22, IBM Corp., Armonk, NY) was used. Results presented as mean ± standard deviation. Differences between groups were determined by independent samples t-test. Correlations between variables were determined by the Pearson correlation coefficient. The impact of possible confounders on the observed correlations was assessed by multivariable regression analysis. Two-tailed *P* values < 0.05 were considered significant.

## Results

### Study groups

The study comprised 76 patients, i.e., 46 subjects categorized as the control (Ctrl) group, in whom the diagnostic assessment left them with a diagnosis of intracranial cyst (arachnoid or pineal), dementia or no identified neurological disease, and 30 individuals denoted as the CSF-group, in whom diagnostic work-up left them with a diagnosis of idiopathic intracranial hypertension (IIH), spontaneous intracranial hypotension (SIH), or hydrocephalus. Table 1 provides demographic information about the two groups as well as information about intracranial volumes, CSF clearance, enrichment of tracer in PSD, and sleep quality.

**Table 1:** The study groups

	Ctrl-group	CSF-group	Statistics
***Demographic***			
Number of participants (n)	46	30	
Sex (Female/Male; n)	34/12	20/10	ns
Age (yrs)	40.8 ± 13.4	45.3 ± 17.0	ns
BMI (kg/m^2^)	27.6 ± 3.9	29.1 ± 5.5	ns
***Blood parameters***			
CRP (mg/L)	2.7 ± 3.1	5.3 ± 5.4	P = 0.02
Hb (g/dL)	13.8 ± 1.1	14.7 ± 5.4	ns
EVF	0.41 ± 0.03	0.41 ± 0.03	ns
Leukocytes (10^9^/L)	7.2 ± 2.4	6.4 ± 1.6	ns
Lymphocytes (10^9^/L)	2.0 ± 0.4	2.0 ± 0.5	ns
Neutrophiles (10^9^/L)	4.0 ± 1.5	3.8 ± 1.2	ns
Platelet number (Thrombocytes; 10^9^/L)	275.0 ± 53.3	264.8 ± 85.9	ns
NLR	2.1 ± 1.0	2.2 ± 1.1	ns
PLR	138.8 ± 24.6	169.4 ± 79.8	ns
***Intracranial volume measures***			
PSD (mL)	4.5 ± 1.9	3.8 ± 2.3	ns
Gray matter (mL)	555.9 ± 46.4	549.3 ± 49.7	ns
White matter (mL)	451.6 ± 54.3	448.7 ± 57.1	ns
CSF volume (mL)	257.1 ± 68.5	343.4 ± 227.9	P = 0.018
Intracranial volume (mL)	1,441.7 ± 126.6	1,485.3 ± 246.3	ns
***CSF clearance parameters***			
T_max_ (h)	7.8 ± 3.6	7.7 ± 3.8	ns
C_max_ (µM)	3.2 ± 1.7	3.4 ± 2.0	ns
T_1/2,abs_ (h)	3.7 ± 2.6	3.7 ± 2.4	ns
AUC_0-∞_ (µM)	63.6 ± 20.6	69.0 ± 21.4	ns
***PSD enrichment***			
3 h (%)	52.9 ± 73.3	28.1 ± 47.3	ns
6 h (%)	126.2 ± 106.5	62.5 ± 103.8	ns
24 h (%)	196.5 ± 166.8	85.7 ± 109.8	P = 0.020
48 h (%)	111.6 ± 79.8	79.0 ± 94.3	ns
***Sleep quality***			
PSQI Total score	8.9 ± 4.1	9.5 ± 4.5	ns
Poor sleep quality (PSQI > 5) (Yes/NO; %)	31/15 (48.4%)	19/11 (57.9%)	ns

Continuous data presented as average ± standard deviation

*AUC*_0-∞_ Area under curve, *BMI* Body mass index, *CRP* C-reactive peptide, *Ctrl* Control, *CSF* Cerebrospinal fluid, *C*_max_ Maximum concentration in blood, *EVF* Erythrocyte-Volume Fraction, *Hb* Hemoglobin, *NLR* Neutrophile-Lymphocyte ratio, *NS* Non-significant, *PLR* Platelet-Lymphocyte ratio, *PSD* Parasagittal dura, *PSQI* Pittsburgh Sleep Quality Index, *T*_1/2,abs_ Half-life of absorption, *Tmax* Time to maximum concentration in blood. Differences between continuous variables were determined by independent samples t-test, and between categorical data as Pearson Chi-square test

### Blood markers versus volume of PSD

**Fig. 3:**
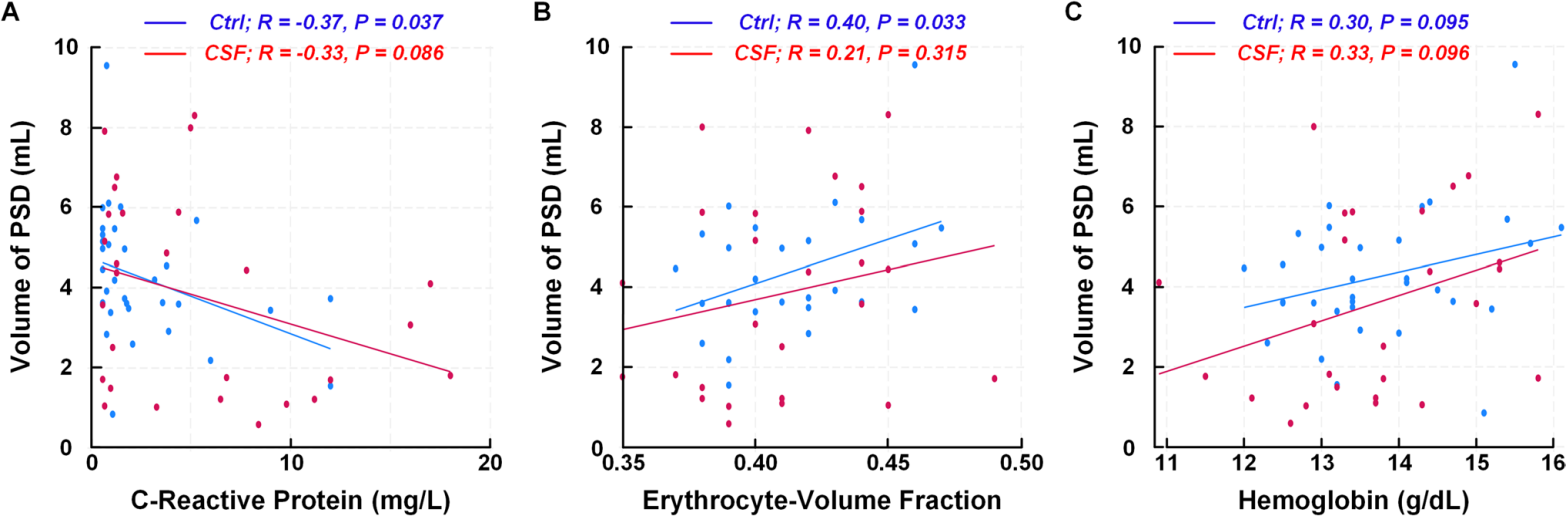
Correlation between volume of parasagittal dura (PSD) and blood markers that may associate with inflammation. The volume of PSD correlated negatively with plasma concentration of C-reactive protein (**A**), while correlated positively with erythrocyte-volume fraction (**B**) and hemoglobin (**C**). Each plot presents the fit line and the Pearson correlation coefficient (R) with *P*-value. Blue line: Ctr-group. Red line: CSF-group

For the total cohort, there was a significant negative correlation between blood CRP concentration and volume of PSD (Pearson correlation −0.36, *P* = 0.005). This was statistically significant for the Ctrl but not the CSF group (Fig. 3A). The erythrocyte volume fraction (EVF) correlated positively with PSD volume in all subjects (Pearson correlation 0.27, *P* = 0.045); this association was significant for the Ctrl but not the CSF group (Fig. 3B). Hemoglobin (Hb) was positively correlated with the volume of PSD for all participants (Pearson correlation 0.33, *P* = 0.010), but was non-significant when considering the two groups separately (Fig. 3C). The other blood markers showed no significant correlations with the PSD volume (data not shown).

Multivariable regression analysis including age, sex, diagnosis and intracranial volumes (Table 1) as confounders confirmed the significant correlation between CRP and the PSD volume, while for EVF and Hb the observed correlations became non-significant when considering these confounders (Table 2).

**Table 2:** Results of multivariable regression analysis for association between PSD volume and blood markers

Volume of PSD (mL)	B	Std. Error	Beta	t	Statistics
**CRP (mg/L)**	−128.2	54.1	−0.284	−2.4	*P* = 0.021
Age	35.8	15.7	0.263	2.3	*P* = 0.026
Sex	17.0	613.9	0.004	0.0	*P* = 0.978
Diagnosis (Ctr/CSF-groups)	−452.6	475.7	−0.113	−1.0	*P* = 0.346
Intracranial volumes	3571.0	1448.0	0.342	2.5	*P* = 0.017
**EVF**	361.5	8874.3	0.005	0.0	*P* = 0.968
Age	56.6	17.1	0.391	3.3	*P* = 0.002
Sex	993.8	718.5	0.225	1.4	*P* = 0.173
Diagnosis (Ctr/CSF-groups)	−1189.5	473.4	−0.294	−2.5	*P* = 0.015
Intracranial volumes	2780.7	1463.7	0.271	1.9	*P* = 0.063
**Hb (g/dL)**	185.2	245.7	0.107	0.8	*P* = 0.454
Age	35.9	16.5	0.259	2.2	*P* = 0.034
Sex	188.3	684.7	0.045	0.3	*P* = 0.784
Diagnosis (Ctr/CSF-groups)	−1060.7	459.7	−0.269	−2.3	*P* = 0.025
Intracranial volumes	3528.9	1460.4	0.347	2.4	*P* = 0.019

*B* unstandardized coefficient, *Std. Error* standard error, *Beta* standardized coefficient (may be interpreted as a correlation coefficient), *P-value*, P value from model

There were no significant correlations between the blood markers and the other intracranial volume measures presented in Table 1 (data not shown).

### Blood markers versus measures of CSF clearance

Tracer enrichment within PSD is a possible proxy of meningeal lymphatic clearance at vertex. There was a significant positive correlation between leukocyte levels in blood and tracer enrichment at PSD after 6 h in the CSF-group (*R *= 0.61, *P* = 0.023), but no other significant correlations between the blood markers and CSF tracer enrichment in PSD at 3, 6, 24 or 48 h in the Ctrl or CSF groups (data not shown).

**Fig. 4:**
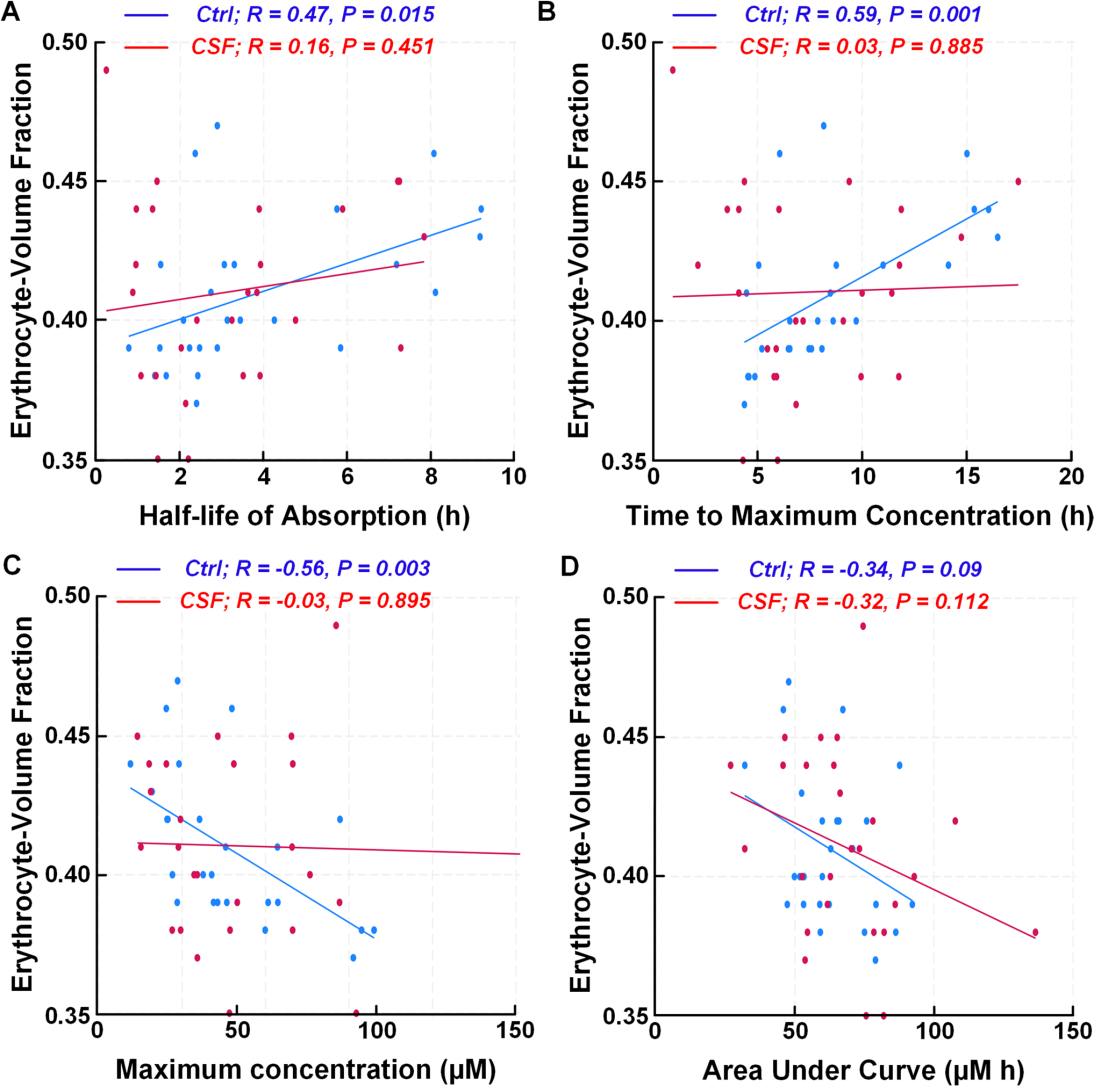
Correlation between blood-based CSF clearance variables and erythrocyte-volume fraction (EVF). The erythrocyte-volume fraction (EVF) correlated positively with CSF half-life of absorption (**A**), time to maximum concentration (**B**) and negatively with maximum concentration (**C**) and area under curve (**D**). Each plot presents the fit line and the Pearson correlation coefficient (R) with *P*-value. Blue line: Ctr-group. Red line: CSF-group

In all participants, there was a positive correlation between EVF and half-life of absorption of the CSF tracer (*R* = 0.30, P = 0.033), which was significant in the Ctrl group, but not the CSF group (Fig. 4A). Furthermore, in the Ctrl group EVF correlated significantly with time to maximum concentration (Fig. 4B), maximum concentration of tracer in blood (Fig. 4C), but there was no significant correlation with area under curve (Fig. 4D). The multivariable regression analysis indicates that these correlations may largely be explained by sex (Supplementary Table 1).

### Blood markers versus subjective sleep quality

For the entire cohort, there was a positive correlation between higher CRP and global PSQI score (higher scores indicative of impaired subjective sleep quality; *R* = 0.34, P = 0.011), as well as between CRP and the PSQI sub-categories subjective sleep quality (*R* = 0.28; *P* = 0.041), sleep duration (*R* = 0.42, P = 0.002) and use of sleep medication (*R* = 0.26, *P* = 0.053; Pearson correlation coefficient). Further analysis showed that the correlation between CRP was significant for the CSF group (global PSQI score *R* = 0.46, *P* = 0.023; subjective sleep quality *R* = 0.50, *P* = 0.14; sleep duration *R* = 0.56, *P* = 0.005; and use of sleep medication *R* = 0.43, *P* = 0.35), but no significant correlations were found in the Ctrl group. Neither the global PSQI score nor the PSQI-subcategories were significantly associated with the PSD volume (data not shown).

## Discussion

The main observation of this study is that higher blood concentrations of CRP was correlated with lower volume of PSD, suggesting an association between the size of this anatomical structure and systemic inflammation.

Increasing CRP may be indicative of non-specific systemic inflammation, while low blood levels of Hb and EVF may be associated with inflammation, as shown for various diseases [9, 19, 32]; however, neither Hb nor EVF are categorized as inflammatory markers per se. Both NLR and PLR ratio are two extensively used non-specific inflammatory markers that have been proven very useful in evaluating inflammation in several diseases [22]. For instance, NLR is not only an indicator of infection or inflammation, but in fact may be increased by any cause of physiological stress [22]. In addition, the prompt response may make NLR a better reflection of acute stress than other laboratory values, making it perhaps more useful in acute rather than chronic conditions [22].

We first hypothesized that with increasing inflammatory activity, the volume of PSD would increase. The fact that inflammatory cardinal signs such as edema cause the environment to swell, would make us think that the volume of the PSD would increase as a response to inflammatory activity. Furthermore, increasing PSD volume was associated with impaired clearance of a CSF tracer [23] as well as with increased global cerebral amyloid-β burden [31]. However, opposite of what might be anticipated beforehand, we found that lower volume of PSD correlated with higher blood concentrations of CRP. We are tempted to speculate that lower PSD volume reflects a brain protective compensatory mechanism for inflammation. In comparison, imbalance between the inflammatory response system (IRS) and the compensatory immune response system (CIRS) may cause the cerebral white matter to shrink in patients with schizophrenia (SCZ). The IRS/CIRS ratio in SCZ was significantly higher than that in the healthy controls, and SCZ had a significantly lower whole-brain white matter average [10]. In the present study, we found no significant correlations between the blood markers of inflammation and volumes of gray or white matter.

An alternative explanation of an inverse relationship between inflammation and PSD volume is atrophy caused by chronic inflammation. In line with this assumption, studies from different tissues show that inflammation may be associated with tissue atrophy [2, 15, 20].

Human studies about inflammatory responses of the meninges have mainly been performed in patients with migraine, providing evidence that migraine is accompanied by an inflammatory reaction in the meninges [27]. Ongoing migraine attacks may be accompanied by meningeal edema, and components of the inflammatory response such as vasodilation, oedema and mast cell degranulation can be evoked by electrical or chemical trigeminal nerve stimulation that cause neuropeptide release from perivascular axons innervating the dura mater [27]. From this, it might be expected that systemic inflammation increased the volume of PSD, but this was not found.

To test CSF clearance and the tentative meningeal lymphatic function, we used two approaches, i.e., the imaging measures of tracer enrichment in PSD [23, 28] and the CSF clearance measures [13]. While there was no association between inflammatory parameters and tracer enrichment at PSD, plasma levels of EVF correlated significantly with the blood-based CSF clearance variables. However, this association seems to be explained by confounders such as age, sex and disease. In addition, EVF also may associate with a range of other factors, such as hydration/dehydration, that theoretically could affect CSF clearance.

Considering the two patient cohorts, the Ctrl and CSF groups compared in several respects though significance levels differed between the groups, indicating different relationships in CSF patients. The higher CRP level within the CSF group was largely driven by the higher CRP levels of the IIH subgroup. In the 12 IIH subjects, CRP was higher, which compare with previous reports of elevated inflammatory markers in patients with IIH [12]. Furthermore, CRP was also significantly associated with BMI, which is higher in IIH patients. Previous results showed reduced PSD volume in IIH subjects [23].

Regarding subjective sleep quality, the present observations support an association with systemic inflammation. This finding is in line with previous reports of an association between sleep quality and systemic inflammation [16]. This is of interest regarding the link between sleep disturbance and dementia, including Alzheimer’s disease [17]. The PSD volume was, however, neither associated with the PSQI global score nor the PSQI sub-categories (data not shown).

### Limitations

We acknowledge some limitations with our study. There are relatively few blood biomarkers of inflammation that are used among clinicians, and the presently used blood markers are non-specific when it comes to inflammation. Therefore, these markers cannot alone diagnose a disease, and the markers can be altered from an ongoing inflammatory process unrelated to an active disease, but being related to other variables, for example pregnancy, aging or smoking. However, in this research project, the main aim was not to differentiate between the causes of inflammation, but rather to investigate whether there is evidence of any association between systemic inflammation and morphological structures and functional tasks of the PSD volume, given its proposed role in immunosurveillance.

Another limitation is the heterogeneity of the patient material. The type of CSF disturbance varied among the subjects of the CSF cohort, and in the Ctrl group some degree of CSF alterations cannot be excluded. The latter group did not include healthy subjects since intrathecal MRI contrast was performed on clinical indication only. However, it is important to be aware that both PSD volumes and CSF clearance variables may vary considerably within the same diagnostic group [13, 23]. The comparable trend in correlations between the Ctrl and CSF groups may strengthen the validity of the observations despite inclusion of different diagnostic categories. The significant correlation between CRP and PSD volume was not affected by the disease subtypes as confounders.

We further would comment that definite conclusions about the causation behind the association between CRP and PSD volume cannot be made from these observations. Whether or not systemic inflammation causes a decline in volume of PSD would require consecutive MRI acquisitions. Therefore, future studies are required to determine the mechanistic underpinnings behind the association between systemic inflammation and CSF clearance measures such as PSD tracer enrichment and blood-based CSF clearance measures.

Finally, the blood concentrations of inflammatory markers were obtained at the time gMRI scanning, typically during morning. Only one blood measure of the inflammatory markers was obtained per patient; possible fluctuations during the day were hence not considered.

## Conclusion

The present study disclosed a significant correlation between higher CRP and lower volume of PSD, which indicates that the size of this anatomical structure associates with systemic inflammation. In general, a significant association between variables does not reflect a causal relationship, but we speculate that lower volume of PSD may represent a compensatory response to systemic inflammation. An alternative explanation could be that the PSD volume is undergoing a degenerative process caused by chronic inflammation. Further studies are required to answer whether PSD volume could be a marker of non-specific systemic inflammation.

## Supplementary Information

Below is the link to the electronic supplementary material.

ESM 1 Supplementary Material 1 (PDF 403 KB)

## Data Availability

No datasets were generated or analysed during the current study.

## Non-standard abbreviations and acronyms

CRP: C-reactive protein
CSF: cerebrospinal fluid
EVF: erythrocyte volume fraction
Hb: hemoglobin
IIH: idiopathic intracranial hypertension
Lymph: lymphocytes
MRI: magnetic resonance imaging
Neu: neutrophiles
NLR: neutrophil-to-lymphocyte ratio
PET: positron emission tomography
PLR: platelet-to-lymphocyte ratio
PLT: platelet count
PSD: parasagittal dura
PSQI: Pittsburgh sleep quality index
REF: reference
SIH: spontaneous intracranial hypotension
SSS: superior sagittal sinus
