# Supplementary material for: Volume of parasagittal dura is associated with blood markers of systemic inflammation
Source: Acta Neurochir (Wien). 2025 Sep 25;167(1):255. doi: 10.1007/s00701-025-06682-6 (PMC12464140; doi:10.1007/s00701-025-06682-6)
Supplement: Supplementary file 1 — Supplementary Material 1 (PDF 403 KB) [file 701_2025_6682_MOESM1_ESM.pdf]

Supplementary Material

Volume of parasagittal dura is associated with blood markers of systemic inflammation

Paulina Eide[1] • Erik Melin[2,3,4] • Geir Ringstad[3,4,5,6] • Per Kristian Eide[3,5,7] • Angelika Sorteberg[3,7]

[1]Faculty of Medicine, University of Oslo, Oslo, Norway
[2]Dept. of Radiology, Østfold Hospital Trust, Grålum, Norway;
[3]Institute of Clinical Medicine, Faculty of Medicine, University of Oslo, Oslo, Norway,
[4]Dept. of Radiology, Oslo University Hospital-Rikshospitalet, Oslo, Norway;
[5]K.G. Jebsen Centre for Brain Fluid Research, University of Oslo, Oslo, Norway
[6]Dept. of Geriatrics and Internal medicine, Sorlandet Hospital, Arendal, Norway
[7]Deptartment of Neurosurgery, Oslo University Hospital-Rikshospitalet, Oslo, Norway,

ORCID

Erik Melin	0009-0003-4902-1611
Geir Ringstad	0000-0003-0919-4510
Per Kristian Eide	0000-0001-6881-9280
Angelika G Sorteberg	0000-0003-1076-3696

***Corresponding author:**
Per Kristian Eide
p.k.eide@medisin.uio.no

Supplementary Table 1. Results of multivariable regression analysis for association between EVF and markers of CSF clearance

Erythrocyte-Volume Fraction (EVF)	B	Std. Error	Beta	t	Statistics
**Half-life of Absorption (h)**	0.003	0.002	0.214	1.8	P=0.079
Age	0.000	0.000	0.070	0.6	P=0.574
Sex	0.042	0.010	0.628	4.1	P=0.000
Diagnosis (Ctr/CSF-groups)	-0.003	0.007	-0.051	-0.4	P=0.684
Intracranial volumes	-0.021	0.023	-0.140	-0.9	P=0.362
**Time of Maximum Concentration (h)**	0.001	0.001	0.160	1.3	P=0.208
Age	0.000	0.000	0.051	0.4	P=0.687
Sex	0.042	0.010	0.634	4.1	P=0.000
Diagnosis (Ctr/CSF-groups)	-0.003	0.008	-0.052	-0.4	P=0.686
Intracranial volumes	-0.022	0.024	-0.145	-0.9	P=0.360
**Maximum concentration (µM)**	-0.001	0.002	-0.066	-0.5	P=0.612
Age	0,.000	0.000	0.077	0.6	P=0.551
Sex	0.042	0.010	0.634	4.0	P=0.000
Diagnosis (Ctr/CSF-groups)	-0.005	0.008	-0.076	-0.6	P=0.550
Intracranial volumes	-0.019	0.024	-0.123	-0.8	P=0.444
**Area Under Curve (µM h)**	0.000	0.000	-0.192	-1.5	P=0.149
Age	0.000	0.000	0.098	0.8	P=0.440
Sex	0.041	0.010	0.619	4.0	P=0.000
Diagnosis (Ctr/CSF-groups)	-0.003	0.008	-0.054	-0.4	P=0.667
Intracranial volumes	-0.026	0.024	-0.172	-1.1	P=0.287

B: unstandardized coefficient, Std. Error: standard error, Beta: standardized coefficient (may be interpreted as a correlation coefficient), P-value: P value from model.
